# Unveiling the Spectrum of Ophthalmic Manifestations in Nutritional Deficiencies: A Comprehensive Review

**DOI:** 10.7759/cureus.50311

**Published:** 2023-12-11

**Authors:** Siddhant P Murkey, Akash Agarwal, Pranam Pandit, Sunil Kumar, Arpita Jaiswal

**Affiliations:** 1 Medicine, Jawaharlal Nehru Medical College, Datta Meghe Institute of Higher Education and Research, Wardha, IND; 2 Surgery, Jawaharlal Nehru Medical College, Datta Meghe Institute of Higher Education and Research, Wardha, IND; 3 Obstetrics and Gynaecology, Jawaharlal Nehru Medical College, Datta Meghe Institute of Higher Education and Research, Wardha, IND

**Keywords:** public health campaigns, prevention, essential nutrients, eye diseases, nutritional deficiencies, ocular health

## Abstract

This comprehensive review explores the intricate relationship between nutrition and ocular health, focusing on the crucial roles of essential nutrients like Vitamin A, Vitamin B1 (thiamine), Vitamin B12, Vitamin C, Vitamin D, Vitamin E, Zinc, and Folate (Vitamin B9) in maintaining eye well-being. Nutrient deficiencies have significant consequences, leading to various eye-related issues, from night blindness to age-related conditions such as cataracts and macular degeneration. It is imperative to address these deficiencies, emphasizing the importance of a well-rounded diet with the necessary nutrients. When necessary, supplementation and regular eye examinations are vital components for effectively monitoring ocular health. Public health campaigns and educational initiatives also play a key role in raising awareness about the profound impact of nutrition on eye health. Future research should explore personalized nutrition plans, nutrigenomics, longitudinal studies, and targeted nutritional interventions. Such investigations will not only enhance our understanding of this crucial connection but also have the potential to reduce the global burden of eye diseases.

## Introduction and background

The human eye, often considered one of the most intricate and crucial sensory organs, heavily relies on a complex interplay of biological processes to sustain health and functionality. Among these critical factors, proper nutrition is pivotal in supporting ocular health and preventing eye-related issues. This comprehensive review delves into the significance of nutrition in safeguarding ocular well-being and explores the intricate relationship between dietary choices and eye health [1].

While nutrition is widely acknowledged as a fundamental aspect of overall health, the importance of specific nutrients in maintaining the integrity of ocular tissues, visual acuity and preventing eye disorders is sometimes overlooked [2]. Inadequate or imbalanced nutrition can result in nutritional deficiencies, profoundly impacting ocular health. Malnutrition and the absence of essential vitamins and minerals can manifest in various eye conditions, affecting vision and, in some cases, leading to permanent damage [3]. This review aims to illuminate the extensive consequences of nutritional deficiencies on the eyes and advocates for increased awareness of the importance of a well-rounded diet in preserving optimal visual function.

The primary objective of this review is to provide a comprehensive exploration of the relationship between nutritional deficiencies and ophthalmic manifestations. It endeavours to compile existing knowledge on the impact of specific vitamins and minerals on ocular health, such as vitamins A, C, D, and E. By examining scientific evidence, clinical case studies, and epidemiological data, this review aims to offer a holistic perspective on the role of nutrition in preventing eye disorders and maintaining clear, healthy vision.

## Review

Nutritional deficiencies and ophthalmic health

Definition of Nutritional Deficiencies

 

Nutritional deficiencies arise when the body lacks a sufficient supply of essential nutrients necessary for proper functioning. These indispensable nutrients comprise vitamins, minerals, and other dietary components crucial for various physiological processes. In the realm of ophthalmic health, such deficiencies can stem from inadequate intake or improper absorption of specific nutrients essential for the eyes' well-being. These deficits can manifest in ocular issues, from minor discomfort to severe vision impairment [4].

The Role of Essential Nutrients in Eye Health

Vitamin A: This essential nutrient, known for its pivotal role in ocular health, is crucial for maintaining clear vision. It is primarily responsible for the health of two vital components of the eye: the cornea and the retina. The cornea's health is critical for maintaining the eye's structural integrity, while the retina's proper functioning, especially in low-light conditions, is essential for night vision. A deficiency in children and adults and a concentration of <0.35 µmol/L indicates severe vitamin A deficiency, making it an indispensable nutrient for preserving the gift of sight [5].

Vitamin C: As a potent antioxidant, vitamin C contributes significantly to the overall health of blood vessels within the eyes. Maintaining the integrity of these blood vessels is crucial for proper blood flow and optimal eye function. Vitamin C's potential to prevent cataracts is also noteworthy [6]. By reducing oxidative stress, considered deficient with serum levels below 11 μmol/L, the development of cataracts, characterized by clouding of the eye's lens, thus contributing to long-term eye health [6].

Vitamin D: Emerging research has shed light on the importance of vitamin D in eye health. It is associated with preventing age-related macular degeneration (AMD), which can lead to central vision loss [7]. Vitamin D's role in reducing eye inflammation is another valuable aspect of its function, making it a potential factor in safeguarding ocular health as we age [7].

Vitamin E: As an antioxidant akin to vitamin C, vitamin E is pivotal in safeguarding eye health through intricate cellular mechanisms. Specifically, vitamin E is instrumental in preserving the integrity of cell membranes within the eyes, a critical aspect of maintaining the overall structure and function of the eyes. The protective function of vitamin E extends to mitigating oxidative damage to these membranes, thereby potentially reducing the risk of developing cataracts [8]. This multifaceted role emphasizes vitamin E's significant contribution to the eyes' long-term health. In the following, we delve into a more detailed exploration of the specific cellular and molecular processes through which vitamin E exerts its protective effects on eye cell membranes.

Zinc: Zinc is an essential trace mineral for ocular health due to its involvement in the functioning of enzymes responsible for various visual processes [9]. It is particularly vital for maintaining the retina's health, the light-sensitive tissue at the back of the eye. Adequate zinc levels of 70 to 250 ug/dl in adults and mild deficiency can manifest clinically when values decrease to 40 to 60 ug/dl. are necessary for producing visual pigments and transmitting visual signals to the brain, underscoring their critical role in preserving clear and sharp vision [9].

Omega-3 fatty acids: These essential fats have gained attention for their potential benefits in eye health, particularly in omega-3 index <4%, reducing the risk of dry eye syndrome [10-12]. Dry eyes can cause discomfort, blurred vision, and irritation, and omega-3 fatty acids may help alleviate these symptoms. Additionally, emerging evidence suggests that omega-3 fatty acids may have a role in preventing AMD, a condition that affects the macula, a part of the retina crucial for central vision. By supporting retinal health and reducing inflammation, omega-3 fatty acids maintain ocular well-being and may reduce the risk of age-related eye diseases [10,13-14].

The Prevalence of Nutritional Deficiencies Globally

Nutritional deficiencies affecting ocular health are not limited to one region or demographic. They are a global concern, with varying prevalence in different populations. Micronutrient deficiencies, especially iron, vitamin A, zinc, iodine, and folate, are prevalent in the developing world, affecting an estimated 2 billion people worldwide [11]. They are a significant contributor to infections and are associated with severe illness and death. The factors contributing to these deficiencies include dietary habits, access to a diverse and nutrient-rich diet, socioeconomic factors, and overall public awareness of the importance of nutrition for eye health [11,15].

Ophthalmic Manifestations of Vitamin A Deficiency

Night Blindness: Night blindness is often the first and most common clinical manifestation of vitamin A deficiency. It is characterized by an impaired ability to see in low-light conditions, such as during the evening or in dimly lit environments. This condition occurs because vitamin A is essential for producing rhodopsin, a pigment in the retina that enables the eyes to adapt to low light. In the absence of adequate vitamin A, the regeneration of rhodopsin is impaired, resulting in difficulties in night vision. Night blindness can significantly impact an individual's ability to function in low-light settings and can be particularly concerning in situations like nighttime driving or when navigating in the dark [16].

Xerophthalmia: Xerophthalmia is a spectrum of eye conditions resulting from prolonged and severe vitamin A deficiency. It includes a range of ocular issues, with dry eyes being an early sign. As the deficiency progresses, it can lead to conjunctival inflammation, manifesting as redness and irritation. In more severe cases, xerophthalmia can progress to the development of corneal ulcers, which are open sores on the cornea. These ulcers can cause intense pain increased light sensitivity, and, if left untreated, may lead to blindness. Therefore, xerophthalmia is a critical indicator of advanced vitamin A deficiency and requires immediate intervention to prevent irreversible vision loss [17].

Bitot's spots: Bitot's spots are small, foamy, white, or greyish spots that appear on the conjunctiva, a thin, transparent membrane covering the white part of the eye. They are a hallmark sign of vitamin A deficiency and are often observed with other ocular issues. These spots indicate a disturbance in the normal maintenance of conjunctival tissue, leading to their characteristic appearance. The presence of Bitot's spots clearly indicates vitamin A deficiency and underscores the need for intervention to prevent the progression of xerophthalmia and other severe eye conditions [18].

Keratomalacia: Keratomalacia is a severe and advanced form of corneal ulceration that can result from prolonged and untreated vitamin A deficiency. The condition involves the softening and deterioration of the cornea, the transparent front part of the eye. This process can lead to severe vision impairment or even blindness. Keratomalacia is a critical concern in regions with high rates of vitamin A deficiency, particularly among children. Its prevention and management require timely and appropriate vitamin A supplementation and nutritional interventions to reverse the damage to the cornea and preserve vision [19].

Diagnosis and Management of Vitamin A Deficiency-Related Eye Conditions

Vitamin A supplementation: In cases of severe vitamin A deficiency, vitamin A supplementation is a critical and immediate intervention. This can be administered orally or through injectable forms of the vitamin, depending on the severity of the deficiency. Supplementation is particularly vital in high-risk populations, such as pregnant women, young children, and individuals in regions with a high prevalence of vitamin A deficiency. It effectively replenishes vitamin A stores in the body and can rapidly address symptoms like night blindness, xerophthalmia, and Bitot's spots. This approach has been instrumental in preventing blindness and reducing the impact of vitamin A deficiency-related ocular conditions [20].

Dietary interventions: Long-term strategies for preventing vitamin A deficiency involve promoting a diet that is naturally rich in vitamin A sources. Foods such as leafy greens, carrots, sweet potatoes, squash, and liver are excellent dietary choices to maintain adequate vitamin A intake. Encouraging individuals to incorporate these foods into their daily meals helps ensure a sustained supply of vitamin A. This approach is precious for maintaining vitamin A levels and preventing deficiency in populations with limited dietary diversity [21].

Public health programs: Public health programs have been instrumental in addressing vitamin A deficiency on a larger scale, especially in low- and middle-income countries where the risk of deficiency-related blindness is significant. These initiatives often focus on distributing vitamin A supplements to at-risk populations, conducting mass supplementation campaigns, and integrating vitamin A supplementation with routine childhood immunization programs. Such programs have reduced the prevalence of vitamin A deficiency and its associated ocular manifestations. They play a crucial role in safeguarding the eye health of vulnerable populations and preventing blindness due to vitamin A deficiency [22].

Vitamin C Deficiency

Collagen synthesis: Vitamin C is indispensable for synthesizing collagen, a fundamental structural protein in the body. In ocular health, collagen plays a vital role in forming the cornea framework, the eye's transparent front surface, and the blood vessels within the eyes. Collagen provides structural support and maintains the cornea's clarity and the integrity of blood vessels, which is essential for normal eye function. A deficiency in vitamin C can lead to weakened collagen, potentially resulting in ocular issues and impaired overall eye health [23].

Antioxidant protection: Vitamin C is a powerful antioxidant in the body, helping protect the eyes from oxidative stress. Oxidative stress occurs when free radicals, highly reactive molecules, damage cells and tissues. The eyes are particularly vulnerable to oxidative stress due to exposure to sunlight and environmental factors. Vitamin C's antioxidant properties help neutralize free radicals, reducing the risk of oxidative damage to ocular tissues. This protective function is crucial for preventing the development of cataracts, characterized by the clouding of the eye's lens, and can be influenced by oxidative stress [24].

Blood vessel health: Proper blood vessel health within the eyes is essential for maintaining adequate blood flow and delivering nutrients to ocular tissues. Vitamin C plays a pivotal role in preserving the integrity of blood vessels, ensuring their strength and flexibility. When blood vessels are healthy, they can effectively transport oxygen and nutrients to the eyes, contributing to optimal eye function. Additionally, maintaining blood vessel health is vital for preventing conditions like diabetic retinopathy, a severe complication of diabetes that affects the blood vessels in the retina. Vitamin C's contribution to blood vessel health underscores its significance in maintaining eye health [25].

Causes and Risk Factors for Vitamin C Deficiency

Inadequate diet: Vitamin C deficiency is frequently the consequence of an inadequate diet. The primary dietary sources of vitamin C are fruits and vegetables, such as citrus fruits, strawberries, bell peppers, and broccoli. When individuals do not consume a variety of these foods regularly, they may not obtain sufficient vitamin C, potentially leading to deficiency. An inadequate diet can concern populations with limited access to fresh produce or highly restricted dietary choices [26-28].

Ophthalmic Manifestations of Vitamin C Deficiency

Dry eyes: Vitamin C deficiency can contribute to the development of dry eyes, a condition characterized by insufficient tears to lubricate the eye's surface. Tears are essential for maintaining ocular comfort, clarity, and health. When there is an inadequate supply of tears, the eyes may become dry and irritated, causing discomfort, redness, and a gritty feeling. Vitamin C's role in maintaining eye health includes supporting the mucous membranes that produce tears. Thus, a deficiency in this essential nutrient can disrupt the normal tear film composition, leading to dry eyes and associated discomfort [29].

Cataracts: Although cataracts have multiple causes, vitamin C deficiency is one of the factors that may increase the risk of developing these cloudy lens opacities. Cataracts occur when the proteins within the eye's lens clump together, leading to impaired vision. Vitamin C, as an antioxidant, plays a critical role in protecting the eye's lens from oxidative damage. Inadequate vitamin C levels can weaken this protective function, potentially making the lens more susceptible to oxidative stress. Over time, oxidative damage can contribute to Cataract formation, highlighting the importance of maintaining sufficient vitamin C levels for long-term eye health [30].

Retinal haemorrhages: Severe vitamin C deficiency can have more serious consequences for the eyes, including retinal haemorrhages. The retina is a susceptible and delicate tissue at the back of the eye responsible for capturing and transmitting visual information to the brain. A lack of vitamin C can weaken the blood vessels within the retina, making them more prone to rupture and causing bleeding within the retina. This condition can significantly affect visual function and impair vision if not promptly addressed [31].

Reduced antioxidant protection: Vitamin C is a potent antioxidant that helps protect the eyes from oxidative damage caused by free radicals. Inadequate vitamin C levels can reduce the eye's ability to counteract oxidative stress, making ocular tissues more vulnerable to damage. This increased vulnerability can raise the risk of various eye disorders and conditions, as oxidative stress is associated with the development and progression of many eye diseases. Maintaining sufficient levels of vitamin C is essential for ensuring the eyes are equipped with the necessary antioxidant defences to preserve their health and functionality [24].

Diagnosis and Management of Vitamin C Deficiency-Related Eye Conditions

Vitamin C supplementation: In cases of vitamin C deficiency or specific eye conditions associated with this deficiency, vitamin C supplementation is a critical therapeutic intervention. These supplements can be administered orally or intravenously, depending on the severity of the deficiency and the individual's needs. Intravenous administration is often used when rapid replenishment of vitamin C is required, such as in cases of severe deficiency or specific eye conditions. These supplements aim to quickly restore vitamin C levels in the body, addressing ocular symptoms and supporting overall eye health [6].

Dietary modifications: Promoting dietary modifications to include a variety of vitamin C-rich foods is an effective preventive and management measure. Encouraging the consumption of citrus fruits (like oranges and grapefruits), berries, leafy greens (such as spinach and kale), and other vitamin C sources can ensure a sustained supply of this essential nutrient in the diet. Dietary modifications are particularly valuable for individuals at risk of deficiency or mild deficiencies, as they can help maintain adequate vitamin C levels and prevent further ocular issues [32].

Lifestyle changes: For individuals at risk of vitamin C deficiency due to lifestyle factors such as smoking or excessive alcohol consumption, addressing these habits is essential in preventing further deficiency. Smoking, in particular, can significantly increase the metabolic demand for vitamin C, necessitating higher intake. By quitting smoking and reducing alcohol consumption, individuals can mitigate the increased risk of vitamin C deficiency and its associated ocular and health implications. Lifestyle changes contribute to maintaining optimal vitamin C status and overall eye health [33].

Management of eye conditions: Eye conditions resulting from vitamin C deficiency, such as cataracts, may require additional ophthalmic interventions. In the case of cataracts, surgical procedures are often necessary to remove the cloudy lens and replace it with an artificial lens, restoring clear vision. While addressing the underlying vitamin C deficiency is vital, managing the eye condition may involve surgery and postoperative care. Addressing the deficiency and providing appropriate ophthalmic interventions ensures comprehensive ocular health management and associated conditions [30].

Vitamin D deficiency

Vitamin D and its Role in Maintaining Healthy Eyes

Regulating calcium: Vitamin D is critical in maintaining calcium balance in the eyes and is essential for normal cellular function and signalling. Adequate calcium levels are crucial for several ocular processes, including muscle contractions and neurotransmission in the eye and maintaining the proper balance of fluids within the eye. When vitamin D levels are sufficient, the eyes can effectively manage calcium, ensuring these essential processes occur without disruption. This regulation of calcium contributes to overall eye health and functionality [34].

Reducing inflammation: Vitamin D possesses anti-inflammatory properties that can be advantageous for protecting the eyes from chronic inflammatory conditions. Inflammation can damage ocular tissues and exacerbate eye diseases. Vitamin D's anti-inflammatory effects may help mitigate inflammation within the eyes, reducing the risk of conditions like uveitis or dry eye syndrome. By suppressing excessive inflammation, vitamin D supports ocular comfort and long-term health [35].

Modulating the immune system: Vitamin D influences the immune system, playing a role in modulating its activity. This modulation can have implications for the eyes, potentially reducing the risk of autoimmune eye diseases. The immune system mistakenly targets and attacks healthy eye tissues in autoimmune conditions. Adequate vitamin D levels can help regulate the immune response, reducing the likelihood of these autoimmune reactions and preserving eye health [36].

Preventing age-related macular degeneration (AMD): Some research suggests that vitamin D may be associated with a decreased risk of age-related macular degeneration (AMD), a common cause of vision loss in older adults. AMD involves damage to the macula, a part of the retina responsible for central vision. Vitamin D's potential to reduce the risk of AMD is thought to be related to its anti-inflammatory and antioxidant properties. By limiting inflammation and oxidative stress in the eyes, vitamin D may help protect the macula from damage and slow the progression of AMD, contributing to a lower risk of vision loss in older age. However, more research is needed to establish a definitive link between vitamin D and AMD prevention [7].

Causes and Risk Factors for Vitamin D Deficiency

Inadequate sunlight exposure: Limited exposure to sunlight is a significant factor in developing vitamin D deficiency. Sunlight is required for the skin to synthesize vitamin D, and regions with long winters, extended periods of cloud cover, or individuals with sedentary indoor lifestyles can experience insufficient sunlight exposure. In these cases, the skin may not produce enough vitamin D, increasing the risk of deficiency. Adequate outdoor activities and sun exposure, particularly during sun-rich seasons, are essential to prevent deficiency [37-38].

Dietary insufficiency: A diet lacking in vitamin D-rich foods can contribute to deficiency. Vitamin D is found in limited natural food sources, such as fatty fish (e.g., salmon and mackerel), fortified dairy products, and egg yolks. Individuals who do not regularly consume these foods, especially those with dietary restrictions or specific preferences, may need more vitamin D from their diet. Dietary insufficiency is particularly relevant in populations with limited access to such foods [26].

Age: Aging can be associated with an increased risk of vitamin D deficiency. After sun exposure, older adults may experience reduced skin capacity to synthesize vitamin D. Additionally, they may be less active outdoors, limiting their potential for sun exposure. These age-related factors can contribute to a decline in vitamin D levels, making older adults more susceptible to deficiency [39-40].

Ophthalmic Manifestations of Vitamin D Deficiency

Dry eyes: Vitamin D deficiency can contribute to dry eye syndrome, a common ocular condition characterized by insufficient tear production and unstable tear film. Inadequate tear production can lead to dryness, discomfort, redness, and irritation of the eyes. Vitamin D is believed to play a role in regulating the immune response on the eye's surface. In deficiency, this regulation may be impaired, leading to inflammation and reduced tear production, resulting in the symptoms of dry eye syndrome [41].

Uveitis: Uveitis is characterized by inflammation of the uvea, the middle layer of the eye that contains the iris, ciliary body, and choroid. While the exact causes of uveitis are multifactorial, some research has suggested an association between vitamin D deficiency and an increased risk of uveitis. The anti-inflammatory properties of vitamin D may be relevant in mitigating uveitis, and insufficient vitamin D levels can lead to an imbalance in the immune response, contributing to the development of this condition [42].

Autoimmune eye diseases: Some studies have explored a potential link between vitamin D deficiency and autoimmune eye diseases, such as uveitis and scleritis. Autoimmune conditions occur when the immune system mistakenly attacks healthy tissues, including the eyes. Vitamin D influences the immune system; a deficiency may disrupt immune regulation [43]. This disruption can increase the risk of eye autoimmune reactions, potentially triggering or exacerbating these conditions [43].

Age-related macular degeneration (AMD): While the relationship between vitamin D deficiency and age-related macular degeneration (AMD) is complex and not fully understood, evidence suggests that low vitamin D levels may increase the risk of developing AMD. AMD is characterized by damage to the macula, a region in the retina responsible for central vision. The exact mechanisms are still under investigation, but vitamin D's anti-inflammatory and antioxidant properties are thought to protect the macula from oxidative stress and inflammation. Therefore, insufficient vitamin D levels may weaken this protective function and increase AMD risk, particularly in older adults. Further research is necessary to establish a definitive link between vitamin D deficiency and AMD [44].

Diagnosis and Management of Vitamin D Deficiency-Related Eye Conditions

Vitamin D supplementation: Healthcare providers often prescribe vitamin D supplements in cases of vitamin D deficiency. These supplements can be administered orally or intravenously, depending on the severity of the deficiency and the individual's specific needs. Vitamin D supplementation is crucial for rapidly replenishing vitamin D levels in the body and addressing ocular and systemic symptoms associated with deficiency [45].

Sunlight exposure: Encouraging safe and adequate sunlight exposure is a natural and effective way to boost vitamin D levels in low-risk individuals. Sunlight is essential for the skin's synthesis of vitamin D. However, it's important to emphasize the need for responsible sun exposure to minimize the risk of skin damage and skin cancer. Recommendations for safe sun exposure may include spending time outdoors during non-peak UV hours, wearing sunscreen, and ensuring enough skin is exposed to sunlight [46].

Dietary modifications: Promoting dietary modifications to include a variety of vitamin D-rich foods is an essential preventive measure against deficiency. Fatty fish like salmon and mackerel, fortified dairy products, and egg yolks are valuable dietary sources of vitamin D. Encouraging individuals to incorporate these foods into their diet can help maintain adequate vitamin D levels, particularly in populations where dietary diversity may be limited [26].

Ophthalmic interventions: In cases where eye conditions have resulted from vitamin D deficiency, specialized ophthalmic interventions may be required. Depending on the specific eye condition, treatment options may include anti-inflammatory medications, immunosuppressants, or other therapies tailored to address the underlying cause of the ocular issue. Ophthalmologists play a critical role in diagnosing and managing eye conditions related to vitamin D deficiency, and the treatment approach will be based on the nature and severity of the condition [47].

Vitamin E deficiency

Vitamin E and its Role in Ocular Health

Antioxidant protection: Vitamin E is a potent antioxidant that plays a critical role in safeguarding the cells and tissues of the eye from damage caused by free radicals and oxidative stress. The eye is highly susceptible to oxidative damage due to its constant exposure to environmental factors, such as UV radiation and pollutants. Vitamin E's antioxidant properties help neutralize highly reactive free radicals that can harm cell membranes and other eye structures. By preventing oxidative damage, vitamin E contributes to preserving the integrity of cell membranes, thereby maintaining ocular health and functionality [48].

Reduction of inflammation: Vitamin E possesses anti-inflammatory properties that can help mitigate eye inflammation, essential for overall eye health. Inflammation is a natural response to injury or infection, but chronic inflammation can harm ocular tissues. Chronic eye inflammation can lead to uveitis, conjunctivitis, or dry eye syndrome. Vitamin E's anti-inflammatory effects can help alleviate inflammation, reducing discomfort and preventing potential eye damage. In this way, vitamin E contributes to maintaining ocular comfort and long-term health [49].

Prevention of cataracts: Some studies suggest that vitamin E may play a role in reducing the risk of Cataract formation. Cataracts are characterized by the clouding of the eye's lens, which can result in impaired vision. Oxidative stress and the cumulative damage caused by free radicals are associated with Cataract development. Vitamin E's antioxidant properties may help protect the eye's lens from oxidative damage, slowing the progression of cataracts. While more research is needed to establish a definitive link between vitamin E and Cataract prevention, its potential benefits underscore its significance in preserving eye health [50-52].

Causes and Risk Factors for Vitamin E Deficiency

Dietary insufficiency: One of the primary causes of vitamin E deficiency is dietary insufficiency. A diet low in foods rich in vitamin E, such as nuts, seeds, and vegetable oils, can result in insufficient intake of this essential nutrient. Inadequate dietary variety or restrictions can limit access to vitamin E sources. Populations with dietary preferences or restrictions and individuals with limited access to diverse foods may be at a higher risk of deficiency. A balanced diet that includes vitamin E-rich foods is essential to maintain adequate levels of this nutrient [26].

Malabsorption issues: Conditions that affect the absorption of dietary nutrients, such as fat malabsorption disorders, can hinder the uptake of vitamin E. For example, cystic fibrosis, a genetic condition affecting mucus production, can lead to fat malabsorption. Liver diseases and conditions that impair the production of bile, which is essential for fat digestion, can also contribute to malabsorption. When fats are not properly absorbed, the body may have difficulty absorbing fat-soluble vitamins like vitamin E, potentially leading to deficiency [28].

Ophthalmic Manifestations of Vitamin E Deficiency

Retinopathy: Vitamin E deficiency can lead to retinopathy, a condition characterized by damage to the retina, the light-sensitive layer at the back of the eye. The retina is responsible for capturing and transmitting visual information to the brain. In deficiency cases, the retinal cells may become vulnerable to oxidative damage and inflammation, potentially causing vision problems. Retinopathy can manifest as reduced colour perception, difficulty seeing in low-light conditions, and blurred or distorted vision. Vitamin E's antioxidant properties help protect the retina from oxidative stress, and when this protection is compromised, it can lead to retinal damage and associated vision issues [53].

Nystagmus: In severe cases of vitamin E deficiency, individuals may experience nystagmus, characterized by involuntary, rapid, and repetitive eye movements. Nystagmus can significantly impact vision, causing visual disturbances and making maintaining a stable gaze on an object difficult. The exact mechanisms by which vitamin E deficiency leads to nystagmus have yet to be fully understood. Still, it is thought to be related to the disruption of normal eye muscle function and coordination [54].

Muscle weakness: Vitamin E deficiency-related muscle weakness can affect the extraocular muscles that control eye movement. These muscles are responsible for directing the eyes to focus on specific objects. When these muscles are weakened due to deficiency, it can result in strabismus, a condition characterized by misalignment of the eyes. Strabismus can lead to double vision, difficulty focusing on objects, and impaired depth perception. The proper function of these eye muscles is essential for coordinated eye movement and maintaining binocular vision [55].

Decreased visual acuity: Vitamin E deficiency can decrease visual acuity, particularly in low-light conditions. Visual acuity refers to the sharpness of vision and the ability to discern fine details. Inadequate vitamin E levels can impair the functioning of photoreceptor cells in the retina, which are essential for detecting light and transmitting visual information to the brain. As a result, individuals with vitamin E deficiency may experience reduced visual acuity, making it challenging to see clearly, especially in dimly lit environments. Adequate vitamin E is necessary for optimal visual function, and its deficiency can lead to a decline in visual acuity [56].

Diagnosis and Management of Vitamin E Deficiency-Related Eye Conditions

Vitamin E supplementation: Healthcare providers often prescribe vitamin E supplements in cases of vitamin E deficiency. These supplements can be administered orally or intravenously, depending on the severity of the deficiency and the individual's specific needs. Vitamin E supplementation aims to quickly replenish vitamin E levels in the body, addressing ocular symptoms and supporting overall eye health. Intravenous administration may be necessary in severe cases or when individuals have difficulty absorbing oral supplements [26].

Dietary adjustments: Promoting dietary adjustments to include a variety of vitamin E-rich foods is a critical preventive measure against deficiency. Nuts (such as almonds and hazelnuts), seeds (like sunflower and pumpkin seeds), and vegetable oils (including sunflower and safflower oil) are valuable dietary sources of vitamin E. Encouraging individuals to incorporate these foods into their diet is essential to maintain adequate vitamin E levels, especially in populations with dietary restrictions or limited access to diverse foods. A balanced diet with vitamin E helps prevent deficiency and improves overall eye health [57].

Monitoring and ophthalmic care: Regular eye examinations are essential for individuals with ocular issues resulting from vitamin E deficiency. Ophthalmologists play a crucial role in monitoring and managing these conditions. Treatment options may include corrective lenses, vision therapy, or other appropriate interventions to address specific visual impairments. The type of intervention will depend on the nature and severity of the ocular issues and may involve a combination of treatments. Monitoring ensures that any changes in ocular health are promptly addressed and managed to optimize visual function and quality of life [58].

Nutritional deficiencies and age-related eye diseases

Relationship Between Nutrition and Age-Related Eye Diseases

Cataracts: Cataracts are a common eye condition characterized by clouding the eye's natural lens, leading to vision impairment. Nutrition, particularly the intake of antioxidants like vitamins C and E, has been associated with a reduced risk of Cataract formation. These antioxidants help protect the eye's lens from oxidative damage, contributing to Cataract development. A diet rich in fruits and vegetables, which are abundant sources of vitamins C, E, and other antioxidants, may protect against cataracts. Consuming these foods can help maintain the clarity of the eye's lens and reduce the likelihood of Cataract formation. Additionally, a diet of antioxidants supports overall eye health by minimizing oxidative stress and inflammation [59].

Age-related macular degeneration (AMD): AMD is a progressive eye disease that can lead to central vision loss, making it a significant cause of visual impairment in older adults. Nutrition plays a crucial role in reducing the risk of AMD. Specific nutrients, such as lutein and zeaxanthin (carotenoids found in green leafy vegetables like spinach and kale) and omega-3 fatty acids (abundant in fatty fish), have been associated with a decreased risk of AMD. Lutein and zeaxanthin are found in the macula, a region of the retina responsible for central vision, and act as natural pigments that help protect the macula from oxidative damage and harmful light. Omega-3 fatty acids are known for their anti-inflammatory and antioxidant properties, which can help mitigate inflammation and oxidative stress in the macula. Including these nutrients in the diet, either through food sources or supplements, supports the macula's health and reduces the risk of AMD. Moreover, lifestyle choices such as not smoking and managing blood pressure can further contribute to AMD risk reduction [60].

Preventive Measures Through Dietary Choices

Consuming antioxidant-rich foods: Incorporating a variety of fruits and vegetables rich in antioxidants, such as vitamin C, vitamin E, and beta-carotene, into your diet is a fundamental step in promoting eye health. These antioxidants play a crucial role in combating oxidative stress within the eye, which can contribute to the development of cataracts and age-related macular degeneration (AMD). By reducing oxidative damage to ocular tissues, these nutrients help protect the lens of the eye and the macula, ultimately reducing the risk of cataracts and AMD. Antioxidant-rich foods include citrus fruits, nuts, seeds, and colourful vegetables like carrots and sweet potatoes [61].

Eating foods high in lutein and zeaxanthin: Dark leafy greens, such as spinach and kale, are excellent sources of lutein and zeaxanthin, two carotenoids that are known to support macular health and reduce the risk of AMD. These pigments accumulate in the macula and are a natural defence against oxidative damage and harmful light. Including these greens in your diet, whether in salads, smoothies, or other dishes, can help maintain the macula's health and reduce the likelihood of AMD [61].

Including omega-3 fatty acids: Fatty fish like salmon and mackerel are high in omega-3 fatty acids, which possess anti-inflammatory properties and may protect against AMD. These fatty acids help modulate the inflammatory response within the eye and reduce oxidative stress, contributing to AMD development. Including fish in your diet or considering omega-3 supplements can be beneficial in promoting macular health and reducing the risk of AMD [62].

Moderating sugar and refined carbohydrate intake: High sugar and refined carbohydrate consumption can contribute to the development of conditions like diabetes, which is a risk factor for eye diseases such as diabetic retinopathy. Elevated blood sugar levels can damage blood vessels in the retina, leading to diabetic retinopathy and potential vision loss. By moderating your sugar and refined carbohydrate intake and maintaining stable blood sugar levels through a balanced diet, you can reduce the risk of diabetes and the associated eye complications [63].

Balancing diet and weight: Maintaining a healthy weight and blood sugar levels is essential for reducing the risk of developing diabetic retinopathy and other eye conditions associated with diabetes. Obesity and uncontrolled blood sugar levels can contribute to the onset and progression of diabetic retinopathy, which can lead to severe vision impairment. A balanced diet, regular physical activity, and blood sugar management are crucial elements in preserving eye health and minimizing the risk of diabetic eye complications [63].

Nutritional Supplements and Their Role in Preventing Age-Related Eye Diseases

Vitamin and mineral supplements: For individuals at risk of or already experiencing dietary deficiencies, supplements like vitamin C, vitamin E, and zinc can be beneficial in reducing the risk of cataracts and age-related macular degeneration (AMD). These supplements can help bridge the nutritional gap and ensure the body receives the essential nutrients for maintaining eye health. Vitamin C and E are antioxidants that protect the eyes from oxidative stress, while zinc is crucial for the function of enzymes involved in visual processes [64].

Lutein and zeaxanthin supplements: Lutein and zeaxanthin supplements are available and may be considered by those at risk of AMD or individuals with a history of the disease. These carotenoids are known to support macular health and protect against AMD. While these nutrients can be obtained from foods like dark leafy greens, supplements may be recommended when it is challenging to incorporate these foods into the diet or when higher doses are necessary to manage AMD risk [65].

Omega-3 fatty acid supplements: Fish oil supplements containing omega-3 fatty acids may be recommended for individuals with difficulty incorporating fish into their diet. Omega-3 fatty acids have anti-inflammatory and antioxidant properties that support overall eye health and reduce the risk of AMD. These supplements can provide a convenient way to obtain the benefits of omega-3 fatty acids when dietary sources are limited or when specific dosages are required [66].

Multivitamins: Some multivitamins are specifically formulated for eye health and contain a combination of nutrients that support ocular well-being. These supplements typically include a range of vitamins and minerals known to benefit the eyes, such as vitamins A, C, and E, zinc, and others. Multivitamins designed for eye health can be a convenient way to ensure you receive a comprehensive spectrum of nutrients necessary for maintaining good vision and preventing eye conditions. However, it's essential to consult with a healthcare provider before starting any new supplement regimen to determine your needs and avoid potential interactions or excessive intake of specific nutrients [67].

Prevention and education

Strategies for Preventing Nutritional Deficiencies Related to Eye Health

Balanced diet: A balanced diet is a cornerstone of good eye health. Encourage individuals to consume a well-rounded diet that includes a variety of food groups, such as fruits, vegetables, lean proteins, and whole grains. This diverse diet ensures the intake of essential nutrients for ocular health. Each food group provides unique vitamins, minerals, and antioxidants that play vital roles in maintaining the eyes' structure and function. A balanced diet supports good vision and overall well-being [68].

Nutrient-rich foods: Promote the inclusion of nutrient-rich foods known to benefit eye health. Leafy greens like spinach and kale, colourful fruits and vegetables rich in antioxidants, nuts and seeds, and fatty fish are precious for ocular well-being. These foods provide vitamins and minerals like vitamin C, E, lutein, zeaxanthin, and omega-3 fatty acids that protect against oxidative stress and eye inflammation, reducing the risk of cataracts and age-related macular degeneration [69].

Moderation: Emphasize moderation in consuming sugar and refined carbohydrates. High sugar intake can contribute to the development of diabetes, a significant risk factor for eye diseases like diabetic retinopathy. Encouraging individuals to moderate their consumption of sugar and refined carbohydrates supports stable blood sugar levels and minimizes the risk of diabetes and its associated eye complications [70].

Sun exposure: Advocate for safe sun exposure to promote vitamin D synthesis, as vitamin D plays a role in maintaining eye health. Adequate vitamin D levels are essential for ocular function and reducing the risk of age-related macular degeneration. However, it's crucial to stress the importance of UV-protective eyewear, such as sunglasses, when exposed to intense sunlight. These accessories shield the eyes from harmful UV radiation, which can cause damage to ocular tissues and contribute to eye conditions like cataracts and pterygium [37].

Regular eye examinations: Underscore the importance of routine eye examinations for early detection and management of eye conditions related to nutrition or other factors. Regular eye check-ups can help identify issues like cataracts, glaucoma, or macular degeneration in their early stages when they are more manageable. Monitoring ocular health through eye examinations is vital for preserving and preventing vision loss [71].

Hydration: Promote adequate hydration as it can help maintain tear film quality, reducing the risk of dry eyes. Proper hydration supports the lubrication of the eyes and ensures that they remain comfortable and free from dryness or irritation. Encourage individuals to consume adequate daily water to support overall eye comfort and health [72].

Public Health Campaigns and Education Initiatives

Nutrition awareness: Launching public campaigns that raise awareness about the crucial role of nutrition in eye health and preventing nutritional deficiencies is a fundamental step. These campaigns can use various media channels, including television, radio, social media, and print materials, to educate individuals about the significance of proper eye nutrition. Highlighting the relationship between specific nutrients and eye conditions can empower people to make informed dietary choices that support their ocular well-being [73].

School programs: Introducing nutrition education programs is a proactive approach to instil healthy dietary habits early on. These programs can be incorporated into the curriculum to teach students the importance of a balanced diet, the specific nutrients that benefit eye health, and how to make nutritious food choices. Educating children about nutrition can promote lifelong habits that support overall health and prevent eye-related issues [74].

Community workshops: Conducting community workshops and seminars effectively provides practical guidance on choosing eye-healthy foods and maintaining proper nutrition. These events can be organized in community centres, local healthcare facilities, or schools. They should be interactive and provide individuals with the knowledge and skills to make informed food choices. These workshops can include demonstrations on preparing healthy meals and practical tips for incorporating nutrient-rich foods into daily diets [75].

Targeted initiatives: Developing campaigns tailored to specific at-risk populations is essential for addressing the unique needs of different groups. This includes creating educational materials and outreach efforts specifically designed for older people, children, and individuals with medical conditions that affect nutrient absorption. For example, campaigns for the elderly might focus on preventing age-related eye diseases, while those for children can emphasize the importance of good nutrition for growth and development [76].

Collaboration: Collaborating with healthcare professionals, nutritionists, and eye care specialists is crucial to ensure accurate and up-to-date information is disseminated in educational materials and campaigns. This collaboration can help bridge the gap between eye health's medical and nutritional aspects, providing a comprehensive and evidence-based approach to promoting good vision. Healthcare professionals can also play a vital role in referring individuals to nutrition education programs and workshops when needed, reinforcing the importance of nutrition in eye health [77].

Dietary Recommendations and Guidelines

National dietary guidelines: Encouraging adherence to national guidelines is a foundational step in promoting good nutrition for eye health. These guidelines typically recommend a balanced diet that includes a variety of food groups and emphasizes nutrient-rich foods. By advocating for the adoption of national dietary recommendations, individuals can receive evidence-based guidance on the components of a healthy diet and make informed food choices that support their overall well-being, including eye health [78].

Specific nutrient recommendations: Providing clear recommendations for the intake of eye-beneficial nutrients is essential. This guidance should include daily or weekly nutrient requirements like vitamins A, C, D, E, lutein, zeaxanthin, and omega-3 fatty acids. Clear and actionable recommendations enable individuals to understand their nutrient needs and tailor their diet to meet those requirements, reducing the risk of nutritional deficiencies and eye conditions [79].

Healthy eating plans: Sharing sample meal plans and recipes incorporating eye-healthy foods is a practical way to help individuals follow a balanced diet. These meal plans can illustrate how to include nutrient-rich foods in everyday meals, making it easier for people to adopt eye-healthy dietary habits. By providing recipes and meal ideas that feature fruits, vegetables, lean proteins, and whole grains, individuals can see firsthand how to prepare and enjoy nutritious dishes [80].

Portion control: Educating individuals about portion control is crucial for preventing overeating, which can contribute to obesity and metabolic conditions that affect eye health. Portion control guidance helps people understand appropriate serving sizes and how to avoid excessive calorie intake. This knowledge can support weight management and reduce the risk of conditions like diabetes and risk factors for eye diseases [81].

Supplementation guidance: Providing guidelines on responsibly using dietary supplements is vital. It's essential to emphasize the importance of consulting healthcare professionals before starting any supplementation. Individuals should be educated about when dietary supplements may be necessary, the proper dosages, and potential interactions with medications or medical conditions. Responsible supplementation ensures that individuals receive the proper nutrients without exceeding safe levels, promoting eye health while minimizing potential risks [67].

## Conclusions

In conclusion, this comprehensive review has shed light on the intricate interplay between nutrition and ocular health, underscoring the indispensable roles played by essential nutrients, such as vitamins A, C, D, and E, in sustaining healthy eyes. The review has emphasized the critical necessity of addressing such deficiencies to preserve optimal vision by exploring the consequences of nutritional deficiencies and their potential manifestations in ophthalmic health. The significance of maintaining a well-balanced diet, resorting to nutritional supplementation when necessary, and undergoing regular eye examinations to monitor eye health cannot be overstated. Furthermore, the review has brought attention to the vital role of public health campaigns and educational initiatives in raising awareness about the profound impact of nutrition on eye health. Looking forward, the potential for future research lies in developing individualized nutrition plans, exploring nutrigenomics, implementing longitudinal studies, and applying targeted nutritional interventions. All these endeavours share the overarching goal of preventing eye diseases and safeguarding eye health. Through collaborative efforts, we can collectively enhance the quality of life for individuals while preserving the invaluable gift of sight.
